# Effects of Long-Term Moderate-Intensity Exercise on Autonomic Nervous System Dysfunction Induced by Cardiac Neurovascular Interface Deterioration

**DOI:** 10.31083/RCM40714

**Published:** 2025-10-14

**Authors:** Ruixue Zhao, Jiayi Li, Fengbao Du, Ran Gao, Shuai Zhao, Jiameng Wang

**Affiliations:** ^1^Yan'an University, 716000 Yan'an, Shaanxi, China; ^2^Faculty of Physical Education, Yan'an University, 716000 Yan'an, Shaanxi, China; ^3^Sports Rehabilitation Laboratory, Yan'an University, 716000 Yan'an, Shaanxi, China; ^4^Department of Physical Education, Hainan University, 570228 Haikou, Hainan, China; ^5^International Nursing School of Hainan Medical University, 571199 Haikou, Hainan, China; ^6^Hainan Province Key Laboratory of Sports and Health Promotion, Hainan Medical University, 571199 Haikou, Hainan, China

**Keywords:** long-term exercise, autonomic nervous system, cardiac neurovascular interface, heart rate variability, exercise intervention

## Abstract

Owing to the aging global population, cardiovascular disease (CVD) has become the leading cause of morbidity and mortality worldwide. The aging process is closely associated with cardiac neurovascular interface deterioration, particularly autonomic nervous system (ANS) dysfunction, which has profound effects on cardiovascular health. Recent studies have suggested that long-term moderate exercise can improve ANS function and alleviate CVD risk. This review evaluates the effects of exercise on the cardiac neurovascular interface and ANS function, with a particular focus on the distinct roles of aerobic and anaerobic exercise on cardiac health. Research has shown that exercise significantly enhances heart rate variability, improves autonomic regulation of the heart, and reduces oxidative stress and inflammation, thereby improving cardiac function and reducing the incidence of CVD. Specifically, high-intensity interval exercise and combination training incorporating both aerobic and anaerobic exercise improve the cardiac neurovascular interface and promote cardiac repair. However, while the benefits of exercise are widely recognized, understanding of the factors such as individual differences, exercise intensity, and exercise type needs to be improved to optimize the effectiveness of exercise interventions. Thus, future research should focus on personalized exercise interventions and the identification of biomarkers, such as microRNAs, to enhance the effectiveness of exercise intervention as a clinical treatment strategy.

## 1. Introduction

According to the World Health Organization, the global population of elderly individuals is projected to exceed 1.2 billion by 2025 [1]. Demographic aging is accelerating worldwide. It is estimated that by 2040, individuals aged 60 and older will constitute 19.2% of the global population, with Europe, North America, and Australia/New Zealand experiencing the most pronounced age-structure transitions [2]. Advancing age is correlated with substantially higher rates of age-related morbidity and mortality, notably from cardiovascular diseases and cancer [1, 3]. Moreover, regional trajectories diverge markedly: developed countries already maintain a sizeable proportion of elderly people, while developing countries are experiencing rapid demographic transitions but continue to record lower health-adjusted life expectancy among older cohorts [4, 5, 6]. Aging is closely associated with morphological and functional changes in the cardiovascular system, which are strongly correlated with the incidence of cardiovascular disease (CVD) [7]. CVD remains the leading cause of morbidity and mortality globally, with over 18.5 million people dying from CVD annually, accounting for 31% of global deaths [8].

Studies have observed a close association between cardiac neurovascular interface deterioration and autonomic nervous system (ANS) dysfunction, primarily manifesting as reduced heart rate variability (HRV) and abnormal heart rate recovery. The ANS plays a crucial role in regulating cardiovascular function, and ANS dysfunction can lead to a range of CVDs [9, 10, 11].

Low-intensity exercise (heart rate <140 bpm) induces minimal perturbation of sympathovagal balance, primarily characterized by parasympathetic dominance. Time-domain HRV indices exhibit only transient reductions and typically return to baseline within minutes, indicating rapid restoration of autonomic homeostasis [12, 13, 14]. Moderate-intensity exercise (140 bpm ≤ heart rate ≤ 150 bpm) effectively enhances HRV, boosts parasympathetic activity, and improves cardiovascular and neuroregulatory function [13, 15, 16]. HRV recovery to baseline is achieved within a short period, and cardiac vagal modulation shows its greatest increases under these conditions [13, 16]. In contrast, high-intensity exercise and high-intensity interval training (HIIT) (heart rate ≥150–160 bpm)—and high-intensity exercise induce pronounced sympathetic activation, marked reductions in HRV, and suppression of parasympathetic activity, resulting in prolonged autonomic recovery [12, 13, 15, 17, 18, 19]. During the post-exercise period, elevated heart rate and plasma norepinephrine persist, and HRV remains suppressed for an extended duration [12, 17, 18, 19]. Therefore, moderate-intensity exercise emerges as the optimal approach to enhancing ANS function: It activates cardiac vagal pathways, increases HRV, promotes parasympathetic activity, and avoids the excessive sympathetic activation and delayed recovery inherent to high-intensity exercise [13, 15, 16]. Systematic reviews and numerous experimental studies have shown that moderate-intensity exercise leads to quicker ANS recovery and the most significant improvements in cardiovascular and neural regulation, making it the optimal choice for enhancing ANS function [13, 16]. Exercise intensity and duration are key determinants of post-exercise ANS disruption and recovery rate. High-intensity, short-duration exercise tends to slow ANS recovery [12, 15, 18], while moderate- to low-intensity exercise over an appropriate and sustained period is more beneficial for ANS function and rapid recovery [13, 14, 15, 17, 18]. Strategic periodization of frequency, intensity, and duration can enhance ANS adaptability and improve long-term cardiovascular health.

### 1.1 Differences in ANS Responses Between Acute and Chronic Exercise

The ANS also responds differently to acute (single-session) versus chronic (long-term) exercise. Acute exercise triggers immediate sympathovagal adjustments, typically manifesting as increased sympathetic excitation and vagal withdrawal [20, 21, 22, 23]. In contrast, chronic exercise elicits autonomic plasticity: regular training plays a significant role in enhancing ANS function and reducing cardiovascular risk [21, 23].

### 1.2 Neural Mechanisms of Exercise-Induced Cardiac Autonomic Regulation

During physical activity, cardiac autonomic regulation is achieved through the integrated modulation of sympathetic and parasympathetic outflows to meet the metabolic demands of skeletal muscles [24, 25, 26]. This integration arises from four principal neural circuits: central command, the exercise pressor reflex, the arterial baroreceptor reflex, and cardiopulmonary baroreceptors [23, 24, 27]. Central command from higher brain centers rapidly triggers sympathetic drive and vagal withdrawal; in particular, hypothalamic orexinergic neurons are rapidly recruited during exercise, serving as a key nexus between motor activity and autonomic cardiovascular regulation [28]. In addition, exercise induces upregulation of dopamine β-hydroxylase and oxytocin signaling pathways within autonomic nuclei, fostering synaptic plasticity that enhances parasympathetic tone and baroreceptor reflex function in aged hypertensive animal models [29].

### 1.3 Mechanisms Underpinning Exercise-Induced Modulation of Autonomic Pathways

Physical exercise drives adaptive remodeling of both sympathetic (SNS) and parasympathetic (PNS) outflows, thereby optimizing autonomic balance. With escalating workload, SNS and PNS circuits engage in increasingly coordinated responses, reflected in concurrent shifts in heart rate variability, cutaneous blood flow, and electrodermal activity [15].

### 1.4 Dose-Dependent Effects of Intensity, Duration, and Frequency on Autonomic Recovery

Exercise intensity and duration are the main factors determining the magnitude of autonomic perturbation and the kinetics of post-exercise recovery. High-intensity, short-duration exercise slows down ANS recovery [12, 13, 30], while moderate- to low-intensity exercise elicits more tempered sympathetic responses and accelerates recovery [13, 17, 30, 31, 32]. If the frequency, intensity, and duration of training are periodized appropriately, training fosters greater autonomic adaptability and promotes long-term cardiovascular health.

### 1.5 Autonomic Dysfunction and Neurovascular Interface Degeneration: Aging-Related and Disease-Specific Mechanisms

ANS dysfunction and degeneration of the cardiac neurovascular interface arise both as a function of chronological aging and in response to specific pathologies. With advancing age, cardiac sympathetic and parasympathetic fibers undergo progressive attrition, neurotrophic support wanes, and the neurovascular interface degrades, culminating in heightened arrhythmogenicity and electrical instability—changes underpinned by cellular senescence and oxidative stress pathways [33]. Parallel disease-specific processes exacerbate these deficits: myocardial infarction and cardiomyopathies provoke localized neurotransmitter imbalances and fibrotic remodeling of intramyocardial nerves; malignant arrhythmias and refractory hypertension exhibit sustained sympathetic overdrive and vagal withdrawal; and diabetic autonomic neuropathy—characterized by loss of small-fiber innervation—increases cardiovascular morbidity [34, 35, 36, 37]. Neurodegenerative disorders such as Parkinson’s disease further illustrate how central neuronal loss and peripheral autonomic denervation converge to impair cardiovascular reflexes and hemodynamic stability [38].

In terms of interventions, both moderate-intensity aerobic exercise and HIIT have been shown to improve ANS function, restore sympathetic-parasympathetic balance, reduce cardiovascular risk, and enhance cardiac structure and function, providing potential benefits for patients with cardiovascular diseases [39, 40]. Both modalities mitigate age- and disease-related declines in nerve density and function, improve heart rate variability, and remodel maladaptive neural circuits, thereby reducing arrhythmic risk and augmenting cardiac performance [33, 34, 39, 40].

Exercise promotes cardiovascular health and enhances the heart’s adaptability and repair capacity, thereby improving cardiac function and exercise endurance. A study by Iellamo *et al*. (2019) [11] suggested that endurance training can enhance autonomic control and improve cardiopulmonary function. Moderate exercise is especially important for cardiovascular health, particularly for patients with chronic heart failure, and high-intensity interval exercise (HIIE) has been shown to enhance vagal nerve modulation and reduce the incidence of arrhythmias [41]. Furthermore, the impact of exercise on the cardiac microenvironment has attracted substantial research attention. Studies have shown that exercise regulates specific microRNAs to promote myocardial cell growth and regeneration, thereby aiding in cardiac repair and functional recovery [42, 43].

The role of exercise in cardiac rehabilitation has been widely validated. Giallauria *et al*. (2013) [44] found that early exercise-based cardiac rehabilitation significantly improved myocardial perfusion and left ventricular function while reducing cardiac remodeling. This intervention not only enhanced exercise capacity, but it also improved autonomic function, reducing the risk of arrhythmias [45, 46]. Raczak *et al*. (2006) [47] observed that moderate-intensity endurance training enhanced parasympathetic nervous system activity, while an excessive exercise load increased sympathetic nervous system tone, further emphasizing the positive impact of moderate exercise on the ANS.

Although research has explored the effects of exercise on ANS function, further studies are required to determine whether long-term exercise effectively improves autonomic dysfunction caused by cardiac neurovascular interface deterioration. This research direction not only provides new insights into the prevention and treatment of CVDs, but it also offers potential intervention strategies for improving patients’ quality of life [48, 49, 50]. Daniłowicz-Szymanowicz *et al*. (2011) [51] demonstrated that long-term moderate exercise significantly improved ANS function, providing a basis to further explore the mechanisms underpinning these effects.

## 2. ANS Dysfunction Induced by Cardiac Neurovascular Interface Deterioration

The heart is one of the most vital organs in the human body. It is primarily responsible for delivering oxygen and nutrients throughout the body via the blood circulation, which is necessary to sustain life. However, with aging, the heart gradually deteriorates, and arrhythmias become increasingly common [52, 53]. Numerous studies have shown that aging is accompanied by sustained overactivation of the sympathetic nervous system, which is closely linked to an increase in sympathetic nerve fiber firing rate. This heightened sympathetic activity ultimately leads to a reduction in parasympathetic nervous system activity [45, 54]. The vascular system and the ANS form a complex highly branched network, with both systems functionally dependent on each other. The arrangement of blood vessels and nerves is regulated by neurogenic or angiogenic signals, which modulate the alignment of endothelial cells and nerve fibers, thereby regulating blood vessel and neuronal function and influencing axonal growth. Any imbalance in the function of either of these systems can lead to arrhythmias. Research has shown that there is a close relationship between the incidence of CVDs and sympathetic nervous system activity with aging, with excessive sympathetic activity being strongly associated with CVD onset and progression [55].

Recently, a research team from the German Center for Cardiovascular Research (DZHK) was the first to demonstrate the relationship between ANS dysfunction and aging. With advancing age, the junction between the left ventricular blood vessels and the nervous system in elderly individuals showed neurodegeneration, making it difficult for the heart to regulate heart rate and pulse under stress, ultimately leading to impaired rhythm [33]. Using anti-aging drugs, such as dasatinib and quercetin, the researchers were able to reverse this age-related degeneration, restore heart rate patterns, and reduce electrophysiological instability. Despite these promising results, the effects of aging delay still require further investigation. Many researchers are attempting to directly intervene in the physiological processes that underpin aging [33].

Research suggests that the primary methods for delaying aging are dietary and lifestyle adjustments. To date, anti-aging research has primarily focused on physiological mechanisms, such as inhibiting the nutrient-sensing network, clearing senescent cells, reversing stem cell aging, modulating the microbiome, guiding autophagy, and reducing inflammation [56]. Although some anti-aging substances have shown promising anti-aging effects in animal studies, they still face challenges with regard to their clinical application. For example, rapamycin has been shown to extend the lifespan of Rats by nearly 60% through the inhibition of mechanistic target of rapamycin complex 1 (mTORC1), but it has demonstrated side effects in clinical use [56, 57, 58]. Metformin is thought to extend the lifespan by activating adenosine monophosphate-activated protein kinase (AMPK), regulating the rats gut microbiota, and affecting chromatin, but it has not been proven to extend the lifespan of individuals without diabetes mellitus, and the findings require further validation [59, 60]. Acarbose, spermidine, and non-steroidal anti-inflammatory drugs (such as aspirin and ibuprofen) have also shown lifespan-extending effects in animal experiments, but they exhibit sex differences and side effects, which require further investigation [61, 62, 63, 64]. Other approaches, such as systemic circulatory factor replacement and microbiome modulation, also show potential, but a clear mechanistic understanding of their effects is still lacking [65, 66].

The aging process leads to cardiac neurovascular interface deterioration by regulating the miR-145/semaphorin 3A (Sema3A) axis and cellular senescence [67]. This mechanism not only affects neural density, but it is also closely associated with cardiac dysfunction. Although some anti-aging substances have demonstrated partial anti-aging effects in animal experiments, they still face numerous challenges and controversies with regard to their clinical application. Therefore, identifying and validating new methods for delaying aging remains an urgent priority.

### Autonomic Nervous System Dysfunction, Neurovascular Interface Degeneration, and Aging: Impacts on HRV

Aging is accompanied by progressive attrition of both sympathetic and parasympathetic nerve fibers within the myocardium, resulting in reduced neural density and evident neurodegenerative remodeling of the cardiac neurovascular interface. Concurrently, the endothelial-neural signaling deteriorates, impairing vasomotor control and reducing the heart’s autonomic adaptability.

At the cellular level, senescent cardiomyocytes and vascular cells upregulate axon-repulsive cues, such as Sema3A, which inhibit nerve regeneration and exacerbate the loss of cardiac neural density. Therefore, ANS dysfunction and degeneration of the cardiac neurovascular interface not only reflect normal aging processes but are also tightly linked to pathological conditions, including neurodegenerative and cardiovascular diseases.

These physiological changes manifest functionally as attenuated HRV, decreased parasympathetic activity (e.g., lower root mean square of successive differences (RMSSD)), and imbalances in sympathetic-parasympathetic modulation (e.g., altered low frequency (LF)/high frequency (HF) ratio) [33, 40, 68, 69]. Such abnormalities not only increase the risk of arrhythmias and cardiovascular events but are also associated with heightened systemic inflammation and endothelial dysfunction [68, 70]. The relationship between aging, ANS dysfunction, and HRV metrics is summarized in Table 1.

**Table 1.** **Relationship between aging, ANS dysfunction, and HRV metrics**.

Metric	Full name	Primary indicator	Aging/Disease trend
SDNN	Standard deviation of NN intervals	Overall HRV level	↓ Decreased
RMSSD	Root mean square of successive differences	Parasympathetic activity	↓ Decreased
LF/HF Ratio	Low frequency/high frequency ratio	Sympathetic–parasympathetic balance	↑ Increased or imbalanced

HRV, heart rate variability; ANS, autonomic nervous system.

Notably, experimental clearance of senescent cells can reduce Sema3A expression, promote neural regeneration, increase cardiac nerve density, and improve HRV metrics, thereby reestablishing autonomic equilibrium [33, 69]. As such, various HRV parameters (e.g., standard deviation of NN intervals (SDNN), RMSSD, LF/HF) serve as validated biomarkers for assessing ANS function and overall cardiac health.

## 3. Methods

This narrative review synthesized evidence on how long-term, moderate-intensity exercise, and, by comparison, low- and high-intensity regimens, affect ANS function in the context of the cardiac neurovascular interface degeneration, with particular attention to HRV.

We conducted a systematic search of MEDLINE (*via* PubMed), PEDro, and EBSCO to identify open-access publications (1999–2025) examining the effects of long-term exercise—particularly moderate intensity—on ANS dysfunction associated with cardiac neurovascular interface degeneration. Search terms combined MeSH headings and free-text keywords:

“Long-term exercise” AND “Moderate-intensity”

“Autonomic Nervous System” OR “ANS”

“Cardiac Neurovascular Interface”

“Heart Rate Variability” OR “HRV”

“Exercise Intervention”

After removal of duplicates, 2251 unique records were screened by title and abstract: MEDLINE (906 articles), PEDro (87 articles), and EBSCO (1258 articles).

Full-text articles were assessed against predefined criteria:

Inclusion criteria: (1) Cross-sectional, observational, non-randomized or randomized controlled trials, and reviews; (2) those investigating exercise intensity and/or duration on ANS dysfunction due to cardiac neurovascular degeneration; (3) those reporting at least one HRV parameter (e.g., SDNN, RMSSD, LF/HF ratio).

Exclusion criteria: (1) studies with a sample size of <16 participants; (2) those with duplicate datasets or overlapping cohorts; (3) those with irrelevant outcomes.

## 4. Long-Term Exercise to Improve ANS Dysfunction

ANS dysfunction is closely associated with various health issues, particularly CVD and metabolic syndrome. According to the study by Kingsley and Figueroa (2016) [71], HRV, as a non-invasive assessment method, reflects modulation of the sympathetic and parasympathetic nervous systems, particularly under exercise load, suggesting that changes in HRV can reveal the state of the ANS. For healthy individuals, resistance training has a relatively minor effect on HRV; however, in middle-aged individuals and patients with ANS dysfunction, long-term training improves parasympathetic modulation. Lee *et al*. (2022) [72] found that both resistance training and aerobic exercise effectively improved HRV in middle-aged women, indicating that these two training modalities positively impact ANS activity. Kulshreshtha and Deepak (2013) [73] suggested that exercise interventions improve ANS regulation in patients with fibromyalgia syndrome. Moreover, Lee *et al*. (2003) [74] found that after 2 weeks of endurance training, participants showed significant improvements in HRV, particularly via enhanced parasympathetic regulation.

Recent studies have shown that long-term exercise significantly improves HRV, especially in populations with good cardiovascular health. Amano *et al*. (2001) [75] discovered that after 12 weeks of aerobic exercise training, participants showed significant improvements in HRV, reflecting increased ANS activity, particularly enhanced parasympathetic activity. Moreover, Raczak *et al*. (2006) [47] found that long-term high-intensity training promotes parasympathetic dominance, suggesting adaptive changes in the ANS. In their experiments in mice, Liu *et al*. (2024) [76] observed that aerobic exercise intervention suppressed myocardial cell apoptosis, thereby improving cardiac function, supporting the positive role of exercise in modulating the ANS. Bisaccia *et al*. (2021) [77] indicated that exercise alleviated ANS dysfunction associated with coronavirus disease 2019 sequelae, further demonstrating the broad benefits of exercise for the ANS. However, Herzig *et al*. (2018) [78] showed that HRV changes do not always directly reflect ANS activity, particularly under the influence of cardiac structure and heart rate, which offers a new perspective for understanding the impact of exercise on HRV.

Exercise load directly influences HRV. Wittels *et al*. (2023) [12] demonstrated that an increase in exercise load was negatively correlated with heart rate recovery, suggesting that high-load training may lead to excessive ANS fatigue. In contrast, moderate training loads effectively improved HRV and promoted cardiovascular health [79]. As training progresses, improvements in HRV reflect the enhanced adaptability of the ANS. Vieluf *et al*. (2020) [80] found that an increase in exercise intensity affected multiple aspects of the ANS, indicating that high-intensity exercise may lead to dynamic changes in ANS function.

In summary, long-term exercise training significantly improves HRV and effectively modulates ANS function, playing a crucial role in maintaining the health of the cardiac neurovascular interface. Appropriate exercise load is a key factor in enhancing HRV, helping to prevent health issues caused by ANS dysfunction. Chen *et al*. (2023) [50] highlighted that exercise improves endothelial progenitor cell function in the elderly, positively impacting cardiovascular health and emphasizing the broad benefits of long-term exercise on overall health.

## 5. Impact of Exercise Intervention on the Molecular Mechanisms of Cardiac Neurovascular Interface Deterioration

### 5.1 Aging and Functional Degradation of the Cardiac Neurovascular Interface

Aging leads to functional degradation of the cardiac neurovascular interface, which primarily manifests as a decrease in nerve density and an increase in the expression of the neural repellent factor Sema3A. Sema3A influences nerve axon density by regulating miR-145 expression, potentially leading to instability in cardiac electrical activity [33]. Studies have shown that aging significantly reduces nerve density in the left ventricle, while neural innervation of the right ventricle remains relatively stable between aged and young mice. With aging, the activity of the sensory nerves gradually weakens in mice, with nerve density starting to decline at 16 months of age and further decreasing at 22 months of age [33]. The reduction in nerve density is not caused by a decrease in capillary density; rather, it may be associated with capillary dysfunction and changes in the neural conduits within the vascular system. Moreover, the expression of Sema3A is primarily regulated by vascular cells, and in aging cardiac endothelial cells, the expression of Sema3A and other neural repellent factors is significantly elevated [33, 56, 81].

### 5.2 Impact of Exercise Intervention on the Cardiac Neurovascular Interface

**Fig. 1.**
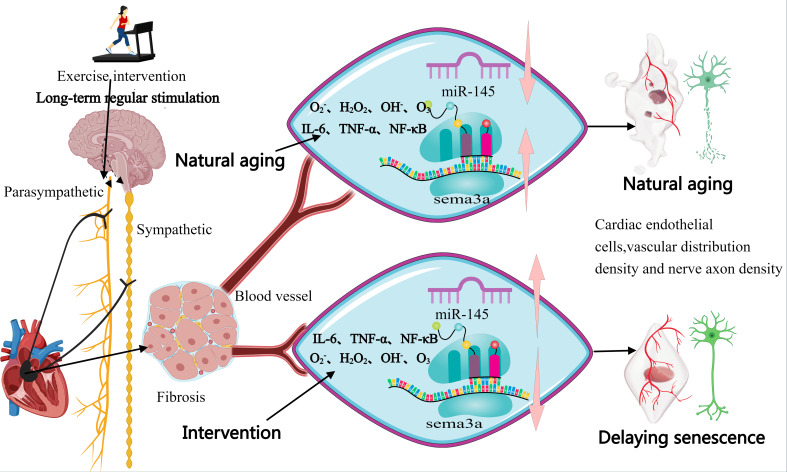
**Hypothesized mechanism underpinning the effects of exercise intervention on cardiac neurovascular interface deterioration**. The pink arrows represent the upregulation or downregulation of gene expression. IL, interleukin; TNF-α, tumor necrosis factor-α; NF-κB, κ light chain enhancer of activated B cells; Sema3A, semaphorin 3A. This figure was created using MedPeer.

Exercise intervention is widely recognized for its positive effects on cardiac health, and it has been shown to improve the activity of the cardiac neurovascular interface. Studies have shown that regular moderate-intensity exercise promotes cardiovascular health and improves the function of aging endothelial cells by modulating oxidative stress (including superoxide anion [O_2_^–^], hydrogen peroxide [H_2_O_2_], hydroxyl radicals [OH^–^], ozone [O_3_], and singlet oxygen [^1^O_2_]) and inflammatory factors (such as tumor necrosis factor-α (TNF-α), interleukin (IL)-1β, interleukin-6, and interleukin-8) [82]. Aerobic exercise promotes myocardial cell renewal; induces cardiac growth; and stimulates the proliferation, migration, and differentiation of endothelial cells, thus achieving endothelial regeneration and angiogenesis. HIIT regulates the expression of Sema3A mRNA, potentially reducing the loss of neural innervation during aging and slowing the progression of neural degeneration (Fig. 1).

#### 5.2.1 The Three Major Energy Systems—Phosphagen System, Glycolytic System, and Aerobic System

The phosphagen, glycolytic, and aerobic energy systems collaborate during exercise to meet the energy demands of the muscles. Each system plays a distinct role during exercise of different intensities and durations [83, 84]. The phosphagen system rapidly provides energy by breaking down stored phosphocreatine and ATP, making it suitable for short, high-intensity activities, such as sprinting and weightlifting [85]. Although this energy supply is quick, its reserves are limited, typically depleting within 10 seconds [86, 87]. The glycolytic system generates energy by anaerobically breaking down carbohydrates into lactate, providing a large amount of energy over a short period. This system is ideal for moderate-duration, high-intensity exercise, such as a 400-meter run [88]. Although this system is an efficient means of providing energy, the accumulation of lactate can lead to muscle fatigue [83, 89, 90]. The aerobic system generates energy by oxidizing carbohydrates and fats, making it suitable for prolonged, low-to-moderate intensity exercise, such as long-distance running and swimming. Although the aerobic system takes longer to activate, it can continuously provide energy to sustain the demands of prolonged exercise [87, 91].

#### 5.2.2 Impact of Phosphagen System Energy Supply on Oxidative Stress and Inflammatory Factors

High-intensity exercise predominantly utilizes the energy supply of the phosphagen system, particularly in HIIE, which induces acute oxidative stress [92, 93]. Although this stress response typically returns to normal within 24 hours, it increases the production of reactive oxygen species, potentially leading to oxidative damage. However, this response also activates redox-sensitive signaling pathways that promote adaptive responses [94, 95]. Additionally, exercise triggers the inflammatory response, marked by an increase in inflammatory cytokines, such as interleukin-6 and tumor necrosis factor-α [96]. This response is generally localized to skeletal muscles and gradually subsides within hours after exercise [97]. The regulation of inflammation after exercise is closely related to the release of anti-inflammatory cytokines, particularly the increase in interleukin-10 [98]. Exercise helps to counteract oxidative stress by increasing the activity of anti-oxidant enzymes (such as superoxide dismutase and glutathione peroxidase) and releasing anti-inflammatory factors from the muscles, as well as alleviating inflammation by downregulating Toll-like receptor signaling pathways [99]. HIIT regulates the expression of Sema3A mRNA, potentially reducing the loss of neural innervation during aging and slowing the progression of neurodegeneration [100, 101].

#### 5.2.3 Impact of Anaerobic Glycolysis on Oxidative Stress and Inflammatory Factors

Anaerobic glycolysis plays a crucial role in oxidative stress and the inflammatory response, particularly with regard to the metabolic reprogramming of immune cells [102]. The glycolytic process occurs under hypoxic conditions and provides energy for inflammatory cells, such as M1 macrophages and T helper 1 lymphocytes, enhancing their pro-inflammatory functions [103, 104, 105]. The inhibition of glycolysis reduces the secretion of pro-inflammatory cytokines, such as tumor necrosis factor-α and interleukin-1β, thereby alleviating the inflammatory response [106, 107, 108]. The metabolic reprogramming of glycolysis is closely linked to the different stages of the inflammatory response, influencing cell activation, proliferation, and differentiation [109, 110]. By inhibiting key glycolytic enzymes, such as glyceraldehyde-3-phosphate dehydrogenase (GAPDH) and hexokinase 2 (HK2), the intensity of the inflammatory response can be effectively reduced [103, 111].

#### 5.2.4 Impact of Aerobic Exercise on Oxidative Stress and Inflammatory Factors

Aerobic and anaerobic exercise each have distinct advantages in improving health. Aerobic exercise significantly reduces oxidative stress and inflammatory factors (such as malondialdehyde, tumor necrosis factor-α, and interleukin-6), while simultaneously increasing the levels of anti-oxidant enzymes (such as superoxide dismutase) and anti-inflammatory factors (such as interleukin-10) [112, 113]. By activating anti-oxidant and anti-inflammatory signaling pathways [such as nuclear factor erythroid 2–related factor 2 (Nrf2) and Janus kinase 2/signal transducer and activator of transcription 3 (JAK2/STAT3)], aerobic exercise effectively mitigates oxidative stress and inflammation, thereby improving overall health [114]. Regular aerobic exercise lowers oxidative markers in the blood and boosts antioxidant factors, with particularly significant effects observed in the elderly population [115, 116, 117].

Anaerobic exercise (such as strength training) also plays an important role in cardiac health [118, 119]. Studies have found that strength training improves the cardiac neurovascular control of the heart, with the effects differing from those of aerobic exercise. In strength training, an increase in exercise load leads to changes in low-frequency and high-frequency HRV indices, indicating that the impact of anaerobic exercise on the ANS is individualized [120]. Anaerobic exercise enhances the adaptability of the cardiac microvasculature, potentially improving overall health by optimizing the cardiac blood supply. Research also suggests that strength training regulates miR-126 to inhibit cardiac fibrosis, thereby improving heart function. Long-term vigorous aerobic training also influences muscle-enriched miRNAs, which play a significant role in cardiovascular adaptation [121].

#### 5.2.5 Exercise Intensity and Individual Differences

The impact of the type and intensity of exercise on oxidative stress and the inflammatory response varies. High-intensity exercise is more likely to induce oxidative stress than moderate-intensity exercise, and it also significantly increases the levels of inflammatory factors, such as interleukin-6 [112, 113]. Furthermore, an individual’s training status and health condition can influence these responses. For example, individuals with obesity typically exhibit higher oxidative stress responses after exercise. Therefore, personalized exercise programs are key to optimizing health benefits. Appropriately selecting the type and intensity of exercise based on the individual health condition of the patient will help to improve their health outcomes [92, 93].

### 5.3 Exercise Interventions for ANS Dysfunction and Neurovascular Degeneration: Clinical Evidence

Accumulating clinical and randomized controlled trial data have demonstrated that targeted aerobic exercise protocols, whether as moderate-intensity continuous training or HIIT, can reverse autonomic imbalance and promote structural integrity of the cardiac neurovascular interface in both aging populations and patients with cardiovascular or neurodegenerative disease. Moderate-intensity continuous aerobic exercise (e.g., 3–5 sessions per week, 30–60 minutes per session, for 12 weeks or longer) has been shown to enhance cardiac vagal activity, improve the sympathetic-parasympathetic balance, reduce cardiovascular risk, and is suitable for cardiac rehabilitation in patients with cardiovascular disease [24, 122].

HIIT protocols (e.g., 2–3 sessions per week, 20–30 minutes per session, with intermittent intensities reaching 85–95% of maximum heart rate, for 8–12 weeks) can also significantly increase HRV, improve ANS function, and exhibit superior time efficiency and cardiovascular adaptability [123, 124]. However, individual HIIT sessions can transiently depress HRV and elevate sympathetic biomarkers, necessitating ≥24 hours of recovery to reestablish baseline autonomic balance [12, 125, 126, 127].

Meta-analyses and systematic reviews further confirm a clear dose–response between exercise “load” (intensity × duration × frequency) and HRV enhancement, moderated by age, baseline fitness, and comorbidities [121]. In older adults, individuals with metabolic syndrome, or post-myocardial infarction patients, individualized moderate-intensity continuous training or HIIT programs have been shown to significantly enhance HRV, reduce sympathetic activity, and improve the function of the cardiac neurovascular interface, thereby contributing to better clinical outcomes [122, 123, 124, 127]. The comparative effects, advantages, and considerations of different exercise interventions are summarized in Table 2 (Ref. [12, 17, 24, 122, 123, 124, 125, 126, 127]).

**Table 2.** **Summary of exercise interventions for ANS regulation and neurovascular restoration**.

Intervention type	Frequency & duration	Physiological effects	Target population	Advantages	Considerations	References
Moderate-intensity aerobic exercise	3–5 times/week, 30–60 min/session, ≥12 weeks	↑ Vagal activity, improved sympathetic/parasympathetic balance, ↓ CVD risk	Patients with cardiovascular disease, older adults	High safety, good sustainability	Requires longer intervention duration for effects	[24, 122]
High-intensity interval training (HIIT)	2–3 times/week, 20–30 min/session, 85–95% heart rate (HR)max intervals, 8–12 weeks	↑ HRV, ↑ ANS function, ↑ cardiovascular adaptability	Healthy adults, metabolic syndrome patients, some elderly	Time-efficient, effective outcomes	Avoid overtraining; recovery period ≥24 hours	[123, 124]
Extreme or excessive high-intensity exercise	Beyond recommended frequency/intensity (e.g., continuous high-intensity)	↓ HRV, ↑ sympathetic activity, short-term ANS suppression	Healthy individuals, athletes	Strong short-term stimulation	Prolonged recovery, potential cardiovascular burden	[12, 17, 125, 126]
Systematic review & meta-analysis findings	Comparative analysis across interventions	Effects depend on duration, frequency, and intensity	Individuals of varying age and health status	Provides evidence-based recommendations	Requires individualized assessment and tailored design	[123, 127]
Personalized intervention (moderate or HIIT)	Designed based on individual status	↑ HRV, ↓ sympathetic activity, improved neurovascular interface function	CVD patients, elderly, metabolic syndrome patients	Personalized, high safety	Requires professional guidance and monitoring	[122, 123, 124, 127]

CVD, cardiovascular disease; HRV, heart rate variability; ANS, autonomic nervous system. ↑ means to increase, and ↓ means to decrease.

#### Effects of Combination Training on the Cardiac Neurovascular Interface

**Fig. 2.**
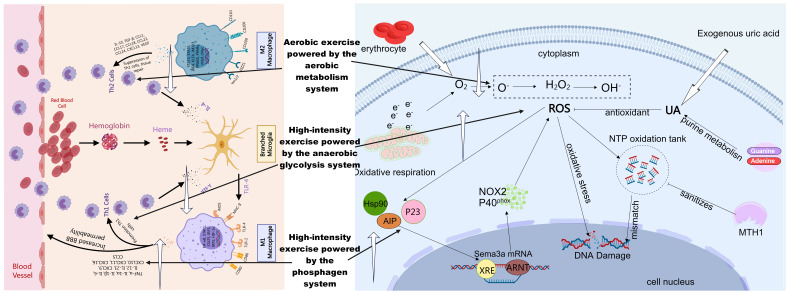
**Hypothesis diagram of the effects of the three energy systems on oxidative stress and inflammatory factors**. The upward white arrows represent promotion, while the downward ones represent inhibition. ROS, reactive oxygen species; AIP, aryl hydrocarbon receptor-interacting protein; ARNT, aryl hydrocarbon receptor nuclear translocator; MTH1, MutT homolog 1, also known as NUDT1; UA, uric acid; Hsp90, heat shock protein 90; NOX2, NADPH oxidase 2; XRE, xenobiotic response element; NTP, nucleoside triphosphate; Sema3A, semaphorin 3A. This figure was created using MedPeer.

Overall, both aerobic and anaerobic exercise have advantages in improving heart health and the cardiac neurovascular interface. Combining aerobic and anaerobic exercise in a comprehensive training program may be the optimal strategy for improving cardiac neurovascular interface function. A study has shown that exercise interventions improve myocardial perfusion and left ventricular function, reducing the negative effects of cardiac remodeling, thus emphasizing the positive effects of exercise on cardiac health [44]. Through appropriate exercise interventions, cardiovascular function can be improved, related gene expression can be regulated, and overall cardiac health can be enhanced. Exercise of different types and intensities affects oxidative stress and the inflammatory response through various mechanisms. In general, aerobic exercise reduces oxidative stress and inflammation, improving health, while high-intensity exercise may increase these responses. Therefore, selecting the appropriate type and intensity of exercise and adjusting them based on the individual health condition of each patient are crucial for maximizing the health benefits (Fig. 2).

### 5.4 A Comprehensive and Personalized Exercise-Prescription System for ANS Dysfunction and Cardio-Neurovascular Interface Degeneration

This precision-medicine system integrates HRV with metabolic, inflammatory, psychological, and lifestyle metrics to (1) stratify individual risk, (2) prescribe tailored exercise “doses”, and (3) dynamically adjust interventions, thereby maximizing autonomic regulation and neurovascular health while ensuring patient safety.

#### 5.4.1 Baseline Profiling: Multidimensional Assessment of ANS Function and Health Status

A rigorous initial evaluation establishes each individual’s physiological, neurovascular, and psychological baseline. The recommended assessment dimensions, tools, and indicators are summarized in Table 3.

**Table 3.** **Baseline multidimensional assessment tools for the autonomic nervous system and related health parameters**.

Assessment dimension	Method/Tool	Indicators/Standards
ANS function	HRV testing (e.g., 24-hour Holter monitor)	SDNN, RMSSD, LF/HF ratio
Cardiovascular function	Resting heart rate, blood pressure, VO₂max	Cardiopulmonary exercise testing (CPET)
Inflammation/Oxidative stress	Serum biomarkers: IL-6, TNF-α, MDA	Closely monitored for high-risk individuals
Body composition & metabolism	BMI, body fat %, insulin resistance, HbA1c	Used to identify metabolic syndrome
Lifestyle & medical history	Questionnaires: physical activity (IPAQ), diet, sleep, medication use	Basis for individual risk stratification
Psychological status	Anxiety/Depression scales (e.g., GAD-7, PHQ-9)	Strongly linked to ANS function

IL, interleukin; TNF-α, tumor necrosis factor-α; HRV, heart rate variability; SDNN, standard deviation of NN intervals; RMSSD, root mean square of successive differences; LF, low frequency; HF, high frequency; MDA, malondialdehyde; GAD-7, generalized anxiety disorder-7; PHQ-9, Patient Health Questionnaire-9; BMI, body mass index; HbA1c, hemoglobin A1c.

#### 5.4.2 Risk Stratification and Exercise Tolerance Analysis

Using the baseline data, individuals were categorized into one of three risk tiers—low, moderate, or high—based on ANS function, physiological metrics, and chronic disease history. This guides exercise intensity recommendations, which are detailed in Table 4.

**Table 4.** **Exercise intensity recommendations based on risk stratification and HRV profiles**.

Risk level	Characteristics	Recommended exercise intensity
Low risk	No cardiovascular/metabolic disease, normal or mildly reduced HRV	Tolerant of moderate to high intensity
Moderate risk	Presence of metabolic syndrome, reduced HRV (↓RMSSD, ↑LF/HF)	Primarily moderate-intensity aerobic exercise, optionally with low-frequency HIIT
High risk	Cardiovascular disease, severely abnormal HRV, elevated inflammation markers	Low intensity with medical monitoring, gradual progression

HRV, heart rate variability; RMSSD, root mean square of successive differences; LF, low frequency; HF, high frequency; HIIT, high-intensity interval training. ↑ means to increase, and ↓ means to decrease.

#### 5.4.3 Personalized Exercise Prescription (Based on FITTP Principles)

Following the FITTP principles—Frequency, Intensity, Time, Type, and Progression—a personalized and evidence-based exercise plan is formulated, as outlined in Table 5.

**Table 5.** **Exercise prescription framework based on the FITTP principle**.

Element	Design content
F (Frequency)	3–5 times per week (adjusted according to risk and tolerance)
I (Intensity)	Moderate intensity (50–70% VO₂max) or HIIT (85–95% HRmax)
T (Time)	30–60 minutes per session (20–30 minutes for HIIT sessions)
T (Type)	Primarily aerobic (e.g., walking, cycling, swimming) + resistance training (1–2 times/week)
P (Progression)	Adjust intensity/type every 4–6 weeks based on HRV improvements

HRV, heart rate variability; HIIT, high-intensity interval training.

#### 5.4.4 Dynamic Feedback and Reassessment Mechanism

To ensure sustainability and safety of the intervention, a dynamic feedback and periodic reassessment mechanism is essential. The recommended monitoring contents and adjustment schedule are summarized in Table 6.

**Table 6.** **Monitoring and adjustment schedule for exercise and autonomic function**.

Frequency	Content
Every 4–6 weeks	Repeat HRV assessments, recheck biochemical markers, test exercise capacity
Real-time	Use wearable devices to monitor heart rate, HRV, fatigue, sleep quality, etc.
Adjustment	Modify exercise intensity and type based on HRV trends to avoid overtraining-induced ANS suppression or HRV decline

HRV, heart rate variability; ANS, autonomic nervous system.

## 6. Conclusion

HIIT and moderate-intensity endurance training improve HRV, regulate oxidative stress, reduce inflammatory factors, and promote heart adaptation and repair. These forms of exercise improve autonomic regulation of the heart, reduce the risk of arrhythmias, and enhance cardiac function and exercise endurance. Furthermore, the regulatory effects of exercise on microRNAs show potential for promoting cardiac cell growth and regeneration, further supporting the recovery of cardiac function.

Although aerobic and anaerobic exercises have different effects on health, combining both in a comprehensive training program may be the optimal strategy for improving cardiac neurovascular interface function. Exercise load, training intensity, and individual differences play significant roles in regulating the ANS and cardiovascular health. Therefore, personalized exercise intervention plans tailored to an individual’s health status and exercise capacity will maximize the health benefits that can be achieved.

Future research should explore the long-term effects of exercise interventions on the ANS. Incorporating modern biotechnology and biomarkers (such as microRNAs) could provide a deeper understanding of the relationship between the ANS and cardiac health, offering more effective guidance for clinical treatment.
